# Supplementary material for: Are carbon emissions trading and green financial instruments synergistic? -Comprehensive quantitative research based on content analysis
Source: PLoS One. 2024 Mar 7;19(3):e0298601. doi: 10.1371/journal.pone.0298601 (PMC10919601; doi:10.1371/journal.pone.0298601)
Supplement: S1 Data — (ZIP) [file pone.0298601.s001.zip › Data/地级市Z_F工作报告.zip!/╣π╢1⁄2/╗▌╓▌╩╨/└┤╫╘╣1⁄2╓┌║┼í╛┬φ┐╦╩2╛▌═°í┐_2015.docx]

各位代表：

　　现在，我代表市人民政府向大会报告工作，请予审议，并请政协各位委员和其他列席人员提出意见。

　　2014年工作回顾

　　2014年，是贯彻落实十八届三中全会精神、全面深化改革的开局之年，也是经济下行压力加大、各种困难明显增多的一年。面对复杂多变的经济形势，在省委、省政府和市委的正确领导下，在市人大和市政协的监督支持下，市政府紧紧围绕尽快进入珠三角第二梯队奋斗目标，突出稳中求进、好中求快、改革创新、率先跨越，全面落实“三大核心任务”，大力实施“六大计划”，全力推动经济社会加快发展，取得了良好成效。

　　（一）经济健康较快发展，转型升级步伐加快。

　　经济实力持续增强。全市生产总值3000.7亿元，比上年增长（下同）10％，用3年时间实现从2000亿元到3000亿元跨越。人均生产总值突破1万美元，达6.37万元，首次超过全省平均水平。地方公共财政预算收入300.6亿元，增长20.2％，用2年时间实现从200亿元到300亿元跨越。固定资产投资1606.7亿元，增长19％。社会消费品零售总额968.7亿元，增长12.3％。进出口总额594.1亿美元，增长3.5％，其中出口363.3亿美元，增长9％。全年新增贷款400亿元，存贷比达71.8％。经济实力稳居全省第五位，尽快进入珠三角第二梯队第一阶段目标如期实现。

　　产业结构不断优化。规模以上工业增加值1543.3亿元，增长13％。电子信息和石油化工两大支柱产业增加值占规模以上工业比重56.8％，先进制造业增加值占规模以上工业比重62％，高新技术产品产值占规模以上工业比重50％。全年接待旅游总人数3968万人次，旅游总收入273.2亿元，分别增长13.7％和20.4％。新引进5家金融机构，组建2家村镇银行和龙门农商行，在全省率先实现县区农商行全覆盖，5家企业在天交所挂牌，4家企业在“新三板”挂牌。服务业实现增加值1160.2亿元，增长7.3％。三次产业比例调整为4.7∶56.6∶38.7，第三产业比重提高1.7个百分点。

　　项目建设扎实推进。在各县区召开推动项目落实现场会，协调解决项目建设用地、审批、融资等难题，推动项目加快建设。189宗重点项目完成投资493亿元，超额完成年度任务。中海油惠炼二期、信利AMOLED等项目加快推进，中海油丙烯酸树脂、比亚迪半导体照明等项目建成投产。2013年以来新立项568宗投资亿元以上项目动工建设385宗。

　　实体经济加快发展。深入实施新“惠28条”和“惠42条”，落实涉企行政事业性收费减免政策，强化企业服务。圆满承办中国手机创新周、粤台经贸合作交流会、第十三届香港珠三角工商界合作交流会，成功举办第三届“云博会”、惠州台湾精品博览会、惠州产品济南展销会和南非展览会，共签订经贸合同214宗，合同金额474亿元。完成“个转企”456家、“小升规”448家。获省新认定高成长性中小企业13家。新增私营企业1.46万户，增长45.9％。TCL集团成为全市第二家年主营业务收入超千亿元企业，新增伯恩光学、惠州比亚迪电子2家年主营业务收入超百亿元企业。

　　创新能力持续提升。全市研发经费支出占GDP比重提高到2.2％。新建国际科技合作、产学研合作创新平台各2家，新认定省级工程技术研发中心9家、高新技术企业42家。国家智慧城市试点全面启动，国家知识产权试点城市建设、国家质量安全示范区创建深入推进，专利授权量增长25％，获国家科技进步二等奖、中国专利优秀奖各1项。引进创新团队10个、领军人才24名，入选国家重点领域创新团队1个，培养和引进高技能人才1.13万人。

　　（二）深化改革开局良好，发展活力不断增强。

　　经济体制改革取得新成效。全面推行工商登记制度改革，内资企业户数、注册资本分别增长42.7％、198.2％。建立“中介超市”平台。公共资源交易制度改革荣获第二届“广东治理创新行政改革奖”。开展全国仲裁“两化”试点和全国反走私诚信体系建设试点。推行产品质量、企业信用“红黑榜”制度，深入开展消费维权、“打假”工作，社会信用体系建设考核排名全省第一，“两建”考核名列全省第二。

　　行政体制改革取得新突破。全面完成市、县区政府机构改革，组建市政府决策咨询委员会。完善政府权责清单，公开45个市直单位307项行政审批事项清单；制订部门服务清单、企业投资管理负面清单。投资项目审批提速80％。完成行政审批标准化建设试点任务。实现网上办事大厅镇街全覆盖，6685项服务事项100％进驻。社会组织发展到3016个。

　　城乡综合改革取得新进展。出台实施主体功能区规划。入选国家新型城镇化综合试点城市。稳妥推进农村土地承包经营权确权登记颁证试点、农村集体建设用地（含宅基地）确权登记发证工作，分别累计确权1.35万户、40.5万户。

　　（三）城乡建设有序推进，基础设施日益完善。

　　规划引领作用进一步加强。完善城市总体规划，修编近期建设规划、轨道交通规划。编制完成土地整治规划、综合运输体系规划、环大亚湾新区空间发展规划、潼湖生态智慧区总体规划。新编制14项控制性详细规划，市中心城区控制性详细规划覆盖率达91％。

　　交通基础设施进一步改善。惠州机场2月5日通航。国际集装箱码头全面竣工。莞惠城轨惠州段建设进展顺利。7条在建高速公路加快推进，惠州海湾大桥（广惠高速东延线）即将通车。圆通桥重建工程如期竣工。完成省道242线、356线水毁修复工程，完成省道路面改造183公里、县道改造31.5公里。新增公交线路22条，新投放公交车282辆。

　　城乡建设管理进一步强化。金山河水清岸绿工程获评中国人居范例奖。完成青年河综合整治工程、潼湖水厂首期工程和3宗小型病险水库除险加固工程、4宗中小河流治理工程。惠州西湖创建国家5A级景区通过景观质量省级评审。新建金山湖公园二期、成语公园、横槎小隐等城市公园，市区新增公共绿地186.1万平方米，城市人均公园绿地面积17.2平方米。实施“三旧0”改造项目70个，面积2366.8亩。违法用地违法建设整治工作扎实推进，数字城管系统初步建成，城市管理水平有效提升。

　　（四）环境保护全面加强，生态建设成效显著。

　　环境质量保持优良。新建成污水处理厂2座、截污管网80公里，城镇污水处理率达95.6％。惠城中心区垃圾分类处理全面推行。“美丽乡村·清洁先行”活动深入开展，城乡生活垃圾无害化处理率达95％以上。淡水河、潼湖流域污染整治继续深化。建成国家级生态镇6个、省级生态镇38个、省级宜居示范城镇2个、省级宜居示范村庄12个。连续七年获全省环保责任考核优秀等级，空气质量排名全国74个重点监测城市第六，第三次获评“中国十佳宜居城市”，入选全国第二批水生态文明城市建设试点。

　　节能减排力度加大。单位GDP能耗下降超额完成省下达任务，53个年度减排项目全面完成。推广应用新能源汽车140辆。清退重污染企业44家，淘汰比例超过10％。淘汰黄标车、老旧车2.65万辆。200家重点企业完成清洁生产审核评估验收。

　　林业生态全面提升。新一轮绿化惠州大行动深入开展，新建生态景观林带213公里、森林碳汇12.8万亩。植物园一期项目建设顺利推进，新增县级以上森林公园3个、森林村庄60个，全市森林覆盖率提高到61.6％，成功创建国家森林城市。

　　（五）区域统筹力度加大，县区经济发展提速。

　　两大平台建设取得新进展。环大亚湾新区石化起步区建设加快推进，惠州南站新城、稔山滨海新城起步区建设全面启动，新建、在建项目27宗，总投资833亿元。潼湖生态智慧区科融新城、国际合作产业园动工建设，新引进企业总部、研发基地、创新孵化等项目20宗，总投资285亿元。

　　县区综合实力进一步增强。惠城、仲恺、博罗等3个县区生产总值超500亿元，龙门、惠东、博罗、惠阳、惠城等5个县区生产总值保持两位数增长，大亚湾、惠阳、博罗、惠城、惠东等5个县区地方公共财政预算收入超30亿元。惠城区服务业比重高达64.2％。惠阳区电子信息产业产值突破300亿元。惠东县成为我市第六个人均GDP超过全省平均水平的县区。博罗县连续七年跻身全国县域经济百强县。龙门县域旅游综合竞争力连续四年位居全省十强。大亚湾石化区位列中国化工园区20强第二。仲恺高新区惠南科创中心获认定国家级科技企业孵化器。深入实施大亚湾开发区对口帮扶惠东县、仲恺高新区对口帮扶龙门县，两个省级产业转移园工业总产值分别增长31％和36.2％。

　　农业农村经济稳步发展。农业实现增加值142.9亿元，增长4.3％。深入实施农业标准化建设，获评省级供港蔬菜质量安全示范区。新增市级农业龙头企业18家、省级名优农产品12个。完成新农村公路改造817公里。在全省率先实现水稻、马铃薯和甜玉米3类农作物免费全参保。为农村居民购买自然灾害公众责任保险。农村住房保险实现全覆盖。

　　（六）惠民举措落到实处，民生质量持续提升。

　　民生投入力度继续加大。全市公共财政民生支出261亿元，增长16％，占公共财政预算支出比重从上年的68.5％提高到70％。基本公共服务均等化综合改革10个领域115个项目全面完成，三年试点任务圆满完成。省民生实事涉及我市31项全部完成年度目标；市十件69项民生实事较好完成。全体居民人均可支配收入22900元，增长10.2％。幸福广东考核主观综合评价、客观综合评价连续两年排全省第一，入选首批“全国民生改善典范城市”，连续五次获评“中国最具幸福感城市”。

　　社会保障功能不断增强。新增城镇就业人员7万多人，转移农村富余劳动力1万多人，城镇登记失业率2.3％。城乡最低生活保障标准提高到每人每月482元。农村“五保”供养、孤儿和城镇“三无”人员保障标准分别提高到每人每月868元、1200元和1300元，达到全省先进水平。企业退休人员基本养老金提高12.7％。城乡居民基本医保财政补助标准提高到340元，6类重特大疾病门诊特定病种的报销比例统一提高到95％。建设保障性安居工程住房3437套，完成城市棚户区改造275户、农村危房（泥砖房）改造4500户。

　　社会福利水平稳步提升。市社会福利服务中心一期建成投入使用。为全市70周岁以上老人购买意外伤害综合保险，年满65周岁在惠居住非户籍老年人免费乘坐公交车。免除殡葬基本服务费用。实施大病医疗慈善救助，为困难群众提供医疗救助托底保障。

　　扶贫开发“双到”成效明显。两年共投入扶贫资金3.94亿元，实施村级扶贫项目1227个，贫困村集体经济收入平均5万元以上；实施贫困户脱贫项目9051个，贫困户人均年收入6000元以上。贫困户医疗保险、社会养老保险参保率达100％。缺乏劳动力的贫困户全部纳入低保。

　　平安惠州建设扎实推进。平安建设十大工程深入实施，全警上路“大巡防”持续开展，“雷霆扫毒”行动取得重大成效，惠东禁毒重点整治顺利“摘帽”，“六大专项”行动考核排名全省第一，“两抢一盗”等违法犯罪案件明显下降，再次获评　“中国最安全城市”。“社会矛盾化解年”各项工作扎实开展，信访维稳成效突出，实现上访批次、人数“双下降”。安全生产监管进一步强化，建成国家首个危险化学品应急救援基地，安全生产形势持续稳定好转。食品药品监管扎实有力，建成覆盖城乡的食品药品安全监管网络。

　　（七）文明创建全面深化，社会事业加快发展。

　　“创文”迎检扎实推进。全国文明城市“三连冠”复检成绩优秀。博罗、龙门获提名全国县级文明城市，博罗园洲镇、罗阳镇田牌村、惠阳沙田镇田头村获提名全国文明村镇，惠州出入境检验检疫局、团市委、惠城区行政服务中心获提名全国文明单位。全市注册志愿者34万人。4人获评“中国好人”。

　　教育发展水平持续提升。新建和改扩建中小学20所，新增中小学学位1.8万个。城乡义务教育生均公用经费补助标准小学提高到950元，初中提高到1550元。新增一所高职院校———　惠州城市职业学院。7个县区通过　“全国义务教育发展基本均衡县”督导评估，创建“广东省推进教育现代化先进市”工作全面推进。

　　文化体育事业更加繁荣。国家历史文化名城申报工作通过专家现场评估考察。文化惠民卡项目进入第二批国家公共文化建设示范项目创建序列。4部作品获省“五个一工程”奖。龙门及惠东平海镇入选“中国民间文化艺术之乡”。稔山镇范和村和多祝镇皇思扬村入选中国传统村落，公庄镇吉水围村、永汉镇鹤湖村等10个村庄入选广东省传统村落。完成6个乡镇、60个自然村的农民体育健身工程，成功举办第五届省少数民族传统体育运动会等大型赛事。

　　卫生计生等社会事业蓬勃发展。市中心人民医院整体改扩建基本完成；市中医医院获评“三甲”；市120急救指挥中心成为全国第2家获世界　“绩优急救中心”资格认证机构。全面推行县级公立医院改革，人均基本公共卫生服务经费标准提高到44元。成功应对H7N9禽流感、登革热、埃博拉疫情。人口计生工作水平进一步提升，全面落实“单独两孩”生育政策。顺利完成我市第三次全国经济普查任务。双拥共建、国防教育、人民防空、老区建设等工作取得新成绩。妇女儿童、老年人、残疾人、红十字等事业取得新发展。外事、侨务、港澳、台务、民族、宗教等工作取得新成效。物价、方志、档案、移民、气象、三防、消防、应急、防震、口岸等工作取得新进步。

　　（八）作风建设深入推进，法治政府建设加快。

　　群众路线教育实践活动扎实开展。紧扣为民务实清廉主题，聚焦“四风”，深入推进教育实践活动，整改完成对市政府领导班子的142条意见建议，健全完善42项规章制度，形成了反“四风”、转作风的长效机制。

　　民主法制建设不断深化。获评全国“六五”普法中期先进市。“法制副主任”制度获广东　“政府治理能力现代化”优秀案例奖。认真执行市人大及其常委会决议，坚持向市人大常委会报告工作、向市政协常委会通报政务，自觉接受人大的法律监督、工作监督和政协的民主监督。办理人大代表议案12件、建议138件，政协提案167件，办复率100％。

　　政府自身建设全面加强。坚持依法行政，在全省首次依法行政考评中获评优秀。严格执行中央八项规定，认真落实国务院“约法三章”，撤销市政府系统议事协调机构132个，精简率47.9％；市直党政机关及参公单位“三公”经费压缩18％。廉洁政府建设扎实推进，审计、监察工作力度加大，市审计局获全国审计机关先进集体称号。政务公开、网络问政不断深化，政府服务质量和效能进一步提升，政府公共服务满意度连续三年排名全省第一。

　　各位代表，过去一年的成绩来之不易。这是省委、省政府和市委总揽全局、正确领导的结果，是市人大、市政协有效监督、大力支持的结果，是全市人民齐心协力、团结拼搏的结果。在此，我代表市人民政府，向辛勤工作在各行各业的广大干部群众，向大力支持政府工作的人大代表、政协委员、各民主党派、工商联和各界人士，向关心帮助地方发展的驻惠部队、武警官兵、驻惠单位，向积极参与惠州建设的港澳台同胞、海外侨胞、国际友人，表示衷心的感谢和崇高的敬意！

　　我们也清醒认识到，经济社会发展长期积累的经济结构不合理问题仍然突出，转方式调结构力度有待加大；体制机制仍然不够健全，全面深化改革亟需持续攻坚；受经济下行压力加大的影响，地区生产总值增速未能实现年度预期目标；创新驱动发展能力不够强，产业转型升级的任务艰巨；区域城乡发展不够平衡，统筹区域城乡发展有待加强；基本公共服务与人民群众期望相比还有差距，改善民生的任务依然繁重；安全生产、食品安全问题仍不同程度存在，社会治理创新尚须深化；政府职能转变仍未到位，行政效率有待提高。对于这些问题，我们一定高度重视，并将采取有力措施，认真加以解决。

　　2015年工作安排

　　2015年是　“十二五”规划的收官之年，是全面深化改革、全面推进法治惠州建设的关键之年，是尽快进入珠三角第二梯队第二阶段的重要之年，做好今年工作意义十分重大。当前，我国经济已经进入以增速换挡、结构调整、动力转换为主要特征的新常态。新常态面临新挑战，更孕育新机遇。国家大力实施　“一带一路”发展战略，加快推进新型城镇化，全面深化改革创新；省委、省政府强力实施珠三角优化发展、粤东西北加快发展和创新驱动发展战略，为我市经济社会持续健康发展提供了有力支持、注入了强大动力。与此同时，我市两大平台建设全面启动，在迈进“高铁时代”后又将跨入“航空时代”，一大批重大基础设施项目、重大产业项目即将建成投产，为我市转型升级、跨越发展提供了有力支撑、奠定了坚实基础。同时，必须清醒看到，当前世界经济总体复苏疲弱态势难有明显改变，国内“三期叠加”阶段仍然面临不少不稳定不确定因素，经济下行压力和潜在风险并存。我们既要增强战略自信，又要增强忧患意识，在省委、省政府和市委的坚强领导下，紧紧依靠全市人民，坚定加快发展的信心，保持专注发展的定力，强化改革发展的措施，努力在新常态下推动新发展、开创新局面，在新起点上再上新台阶、实现新跨越。

　　2015年政府工作的总体思路是：全面贯彻党的十八大、十八届三中、四中全会、中央经济工作会议和习近平总书记系列重要讲话精神，按照省委、省政府和市委的工作部署，主动适应经济发展新常态，坚持“稳中求进、好中求快、改革创新、率先跨越”，继续深入实施“六大计划”，以提高经济发展质量和效益为中心，着力转方式调结构，狠抓改革攻坚，突出创新驱动，统筹城乡发展，推进依法行政，提升工作效率，加强民生保障，确保完成“十二五”规划各项目标任务，迈出尽快进入珠三角第二梯队、建设科学发展的惠民之州更加坚实的步伐。

　　经济社会发展的主要预期目标是：地区生产总值增长10％左右；人均生产总值增长9％左右；固定资产投资增长16％；外贸出口总额增长5％；社会消费品零售总额增长13％；地方一般公共预算收入增长13％；服务业增加值占GDP比重38％；全社会研发经费支出占GDP比重2.3％；居民消费价格累计增长控制在3％以内；全体居民人均可支配收入增长10.5％；城镇登记失业率控制在2.5％以内；常住人口自然增长率、节能减排等各项指标完成省下达任务。

　　为实现上述目标，今年将突出抓好八项重点工作：

　　（一）突出稳中求进，推动经济又好又快发展。

　　始终坚持发展是硬道理，在着力稳增长的同时，不断发展新业态、新技术、新模式，积极培育新增长点，推动经济在新常态下加快发展、提质增效。

　　充分发挥投资的关键作用。坚持实施项目带动发展战略，扎实推进重点项目建设。全年安排重点项目202项，总投资3653亿元，年度计划投资526亿元，其中新开工项目73项，续建项目97项，投产项目32项。大力推进2013年以来新立项投资亿元以上项目建设，不断扩大投资规模、优化投资结构。加大招商选资力度，加强与世界500强企业、跨国集团、大型央企省企务实合作，实现引资质量与数量同步提升。

　　充分发挥消费的基础作用。继续深入开展惠货全球行、全国行、网上行，举办惠州产品澳大利亚展览会、杭州展销会。推进国家信息消费试点市建设，实施一批信息消费试点示范工程，扩大4G信息业务，促进智能终端消费。扶持电子商务发展，力争电商交易额突破1200亿元。培育旅游休闲、文化体育、健康养老等消费热点。推动房地产市场持续平稳健康发展，扩大合理住房消费。深入推进“万村千乡”市场工程，新增“农超对接”连锁店120家。

　　充分发挥出口的支撑作用。深入推进科技兴贸创新基地、外贸转型升级示范基地建设，重点鼓励有自主知识产权和自主品牌的产品出口，进一步优化出口产品结构。积极培育外贸新业态，发展一批服务贸易龙头企业，启动筹建进口商品展销中心。加快推进电子口岸建设，进一步提升通关效率。

　　着力抓好两大平台建设。抓紧编制实施环大亚湾新区产业发展等专项规划，全面推进三大起步区建设，加快跨界基础设施、公共服务设施建设，积极构建特色鲜明的海洋经济产业体系，努力打造引领全市发展的“蓝色引擎”。抓紧上报潼湖生态智慧区总体规划，争取上升为省的发展战略；高起点规划、高标准保护建设潼湖湿地；抓好中韩科技园和新引进的20宗项目建设，推动科融新城、国际合作产业园建设取得实质性进展，努力打造引领全市发展的“绿色引擎”。

　　加大帮扶服务企业力度。深入实施“企业成长计划”，继续抓好“个转企、小升规”工作，加快培育大型骨干企业。创新开展“企业服务月”活动，完善帮扶服务企业常态化机制，落实涉企行政事业性收费减免政策和新“惠42条”。规范运作中小微企业贷款风险补偿基金，切实帮助中小微企业解决融资困难。

　　切实抓好财税金融工作。大力培育新的财税增长点，完善涉税信息交换与共享机制，提高税收征管质量。继续落实财税工作联席会议制度，强化重点税源管理，优化支出结构，推动财政增收节支。新引进3家银行，组建惠阳村镇银行；推动1家企业上市、4家企业在“新三板”挂牌、4家企业在天交所挂牌。积极协调金融机构加大信贷投放，力争新增贷款460亿元。

　　（二）突出创新驱动，不断增强科技创新能力。

　　贯彻落实《关于实施创新驱动发展战略，加快建设创新型城市的意见》，完善科技创新体系，加强企业技术改造，深化产学研合作，推动联合协同创新，促进我市科技创新能力整体提升。

　　着力推动企业技术改造。今年起，每年统筹安排专项资金1亿元用于支持企业技术改造，力争三年完成工业技改投资600亿元，年均增长20％。综合运用财政支持、用地保障、金融服务、企业减负、人才支撑等优惠政策，全力支持企业扩产增效、智能化改造、设备更新、公共服务平台建设和绿色发展。

　　加快建设新型研发机构。完善孵化器和“松绑性”政策，以仲恺高新区科创中心、惠南科创中心为主体，加快建设科技企业孵化器、加速器集群。支持仲恺高新区实施“恺炬创新行动”。支持有条件的企业自建或与高等院校、科研院所、先进园区共建新型研发机构，力争新认定创新平台20家、高新技术企业30家。设立科技创新创业投资基金，促进科技与金融结合。

　　继续深化联合协同创新。加快推动与以色列、乌克兰、白俄罗斯等国家科技合作。积极拓展与中科院自动化研究所、暨南大学、广东工业大学等科研机构、高等院校的协同创新，积极实施重大科技专项，努力取得突破性进展。

　　积极实施知识产权战略。全面加强自主知识产权创造、运用和保护，实施版权保护与版权兴业工程，加快创建全国知识产权示范市。落实资助政策，鼓励企业积极申请专利、注册商标和版权登记，力争专利申请量和授权量分别增长10％。

　　加强创新人才培育引进。启动“天鹅计划”第二批创新团队和领军人才的引进工作，加快实施“万名高技能人才培养引进工程”，力争引进科技创新团队10个以上、科技领军人才30名以上，培养引进高技能人才1万名以上。

　　（三）突出转型升级，着力提升产业综合实力。

　　以产业高端化、集群化、低碳化为目标，进一步做大做强支柱产业、做长做优配套产业、改造提升传统产业，加快打造四大产值超千亿元的产业集群。

　　加快发展石油化工产业。全力推进中海油惠炼二期项目建设，积极谋划中海油惠炼三期项目。加快完善大亚湾石化区基础配套设施。开展精准招商、产业链招商，加快引进石化中下游项目，进一步延伸石化产业链条、壮大产业规模。

　　加快发展电子信息产业。建成信利AMOLED项目一期主体工程等项目。承办第三届中国手机创新周，办好第四届“云博会”。动工建设智能移动终端制造产业园。加快发展新型平板显示、云计算、物联网终端、北斗装备制造等战略性新兴产业。

　　加快发展汽车和新能源产业。抓好东风本田一体化、京兰新能源汽车等项目续建，推动惠阳、博罗、大亚湾三大汽车产业园加快建设。建成亿纬锂电池储能与动力电池系统项目，大力推进LNG电厂热电联产扩建工程、东山海风电厂等项目建设。

　　加快发展现代服务业。力争建成市级现代服务业集聚区25个。深入推进西湖创5A工作，继续抓好环南昆山旅游产业园、巽寮湾滨海旅游度假区建设。大力发展空港、海港经济。加快推进安博物流园、德邦物流园等项目建设。培育发展总部经济、金融物流、商务会展、研发设计、文化创意等新兴服务业。

　　加快改造提升传统产业。支持企业开发新产品、培育新品牌，实现传统产品的换代升级。鼓励企业引进外商投资、先进设备、管理技术，拓展网络营销，从整体上提高传统产业的技术、装备、管理和营销水平，推动我市服装、制鞋、建材等传统产业向产业链高端发展。

　　（四）突出深化改革，全面增强发展动力和活力。

　　坚持市场取向、问题导向和民生指向，狠抓改革攻坚，确保完成今年各项改革任务，让改革红利转化为发展动力。

　　进一步深化经济体制改革。完善宽进严管配套措施，探索实行工商营业执照、组织机构代码和税务登记证“三证合一”登记制度。全面实施投资管理负面清单。深化财税体制改革，贯彻落实新《预算法》，开展建立事权和财政支出责任相适应制度的改革试点。深化投融资体制改革，探索开展政府和社会资本合作等融资模式。深化国有企业改革，大力发展混合所有制经济。深入推进社会信用体系、市场监管体系建设。抓好“中介超市”规范运作。完善公共资源电子化交易运行机制，推进镇级公共资源交易中心标准化建设，力争成为全国改革试点。

　　进一步深化行政体制改革。全面落实政府权责清单、部门服务清单，继续推进行政审批制度改革、行政审批标准化建设。深化投资项目审批制度改革，推进投资项目审批网上全流程办理和信息公开，实现投资审批和备案事项网上办理率达90％。提升网上办事大厅功能，推动网上办事大厅向村居延伸。深化公务用车制度改革。

　　进一步深化城乡综合改革。全面实施主体功能区规划。加快编制实施新型城镇化规划，扎实开展国家新型城镇化综合试点工作。充分利用“三旧”和棚户区改造政策，创新实施统筹规划、综合开发、连片建设。稳妥推进“村改居”工作，加快推进城乡社区“六个一”和农村公共服务站建设。继续抓好农村土地承包经营权确权登记颁证、农村集体建设用地确权登记发证工作。建立农村土地承包经营权流转服务、监管平台。完善粮食生产补贴机制，扩大政策性农业保险实施范围。

　　进一步深化社会治理改革。加快实施社会建设十大行动计划。完善市、县区、镇街三级综治信访维稳中心。扎实开展社会稳定风险评估，切实加强社会风险防范。加大依法管理网络力度，做好网络舆情预警、引导。全面落实镇街领导干部驻点普遍直接联系群众制度，推广“党员带头、社工支撑、义工为辅”的社区社会服务模式，新建社区综合服务中心示范点20个。积极培育发展社会组织，制订政府购买服务标准和规范。

　　进一步深化对外开放合作。积极参与21世纪海上丝绸之路建设，提升开放型经济水平。抓住广东建设自贸区、粤港澳服务贸易自由化的机遇，深化与港澳台交流合作。继续深入实施《珠三角规划纲要》，主动参与深莞惠“3＋2”经济圈建设。在海外设立商务办事处，支持有实力的企业“走出去”，进一步拓展国际市场和发展空间。

　　（五）突出城乡统筹，着力提升新型城镇化质量。

　　以城乡统筹为抓手，以提高质量为关键，更加注重以人为本，更加注重“四化”融合，全面开创我市新型城镇化发展新局面。

　　加强城乡基础设施建设。加快建设市民公园、美术馆、规划展览馆，推进百萃园、鹿江公园和西枝江红楼滩公园建设。完善市区10条连接路。推进市区供水一体化，加快稔平半岛供水工程建设。完成惠东、博罗“中央财政小型农田水利重点县”和博罗“省级水利建设示范县”建设任务。加快推进重点电网工程和天然气管网建设。

　　大力推进交通设施建设。全力确保莞惠城轨惠州段建成。积极推动深惠城轨前期工作。启动建设武深高速惠州段，继续抓好在建高速公路建设，力争建成惠大高速、惠深高速惠州段改扩建、大广高速龙门段、汕湛高速博罗段。加快建设市中心客运枢纽站。积极推进四环路南段、金恺大道、惠新大道、仲恺大道、隆生大桥等路桥建设，完成省道路面改造120公里、县道改造30公里、新农村公路建设503公里。启动建设机场路二期工程。建成华德保税油库及配套码头。

　　积极推动智慧城市建设。落实智慧惠州建设总体规划，建设大数据管理和服务平台，实施大数据应用示范项目，支持云计算、物联网、移动互联网等技术创新和应用。深入推进宽带网络基础设施建设，分批推动免费WiFi覆盖市级公共区域，统筹推进文化、教育、医疗、社保、交通等民生领域信息化建设。

　　努力提高城市管理水平。加快提升数字城管系统功能。抓好流动商贩中心建立试点及推广。坚决整治违法用地违法建设。加快建设城市管线综合管理系统。进一步优化城市路网、交通管理和停车系统，着力解决城市交通拥堵和停车难等问题。大力发展公共交通，积极推动公共交通示范城市建设。

　　加快发展县镇经济。加大对口帮扶力度，推动县区联动协调发展，力争龙门县人均GDP超过全国平均水平。积极推动惠东、博罗、龙门三个县城扩容提质。编制完成建制镇近期建设用地控制性详细规划、行政村村庄规划。继续抓好专业镇公共服务平台建设，促进专业镇加快转型升级。支持县区、镇街规划建设特色产业园区，依托产业园区推动产业集聚发展。

　　扎实做好“三农”工作。加大农业政策扶持和资金投入力度，推广“政银保”支农贷款业务。加强农技创新推广，强化科技兴农。建设高标准农田18.3万亩、现代农业综合示范区6个，特色农产品种植面积稳定在210万亩以上。新增市级以上农业龙头企业10家。在职村干部补贴提高到每人每月3000元，纳入省补助的经济薄弱村办公经费补助提高到每村每年10万元。推进名镇名村建设，抓好博罗省级新农村示范片项目，提升新农村建设水平。

　　（六）突出生态建设，不断提升宜居环境质量。

　　坚持生态优先，坚守生态红线，更加自觉加强生态文明建设，促进环境质量持续优化，加快创建国家生态市。

　　巩固提升“创森”成果。扎实推进林业四大重点工程建设，着力提高造林绿化质量，完善森林生态景观，增强森林碳汇功能。编制环大亚湾森林公园总体规划，新建县级森林公园1个、湿地公园4个，全市森林覆盖率提高到61.9％。

　　深化水生态文明建设。扎实开展全国水生态文明城市建设试点工作，深入实施“南粤水更清”行动计划，动工建设望江沥、新开河、洛塘渠和大湖溪沥综合整治工程，确保完成淡水河流域、潼湖流域污染整治年度任务。落实最严格的水资源保护管理制度，全力确保东江、西枝江水质安全。

　　强化节能减排集约节约用地。深入实施电机能效提升工程，完成企业电机能效改造提升24.7万千瓦，淘汰燃煤锅炉53台。加快推进金山二期、梅湖三期、马安等城镇污水处理设施建设。全面淘汰黄标车、老旧车，完成省下达的推广应用新能源汽车1500辆任务。加快实施大气污染防治行动方案，保持空气质量优良。严格执行耕地“占补平衡”，严守耕地保护红线。加强集约节约用地考核，提高土地利用效率。

　　大力推进生态创建。深入推进“美丽乡村·清洁先行”行动，同步开展“美丽乡村·清水治污”、“美丽乡村·绿满家园”活动，新建农村生活污水处理设施150座以上，建设森林村庄60个。进一步推进垃圾分类工作，新建和升级改造垃圾转运站各5座。加快推进市植物园、海洋生态园、考洲洋生态公园和市环境生态园建设。创建国家级生态乡镇15个、生态村2个，省级生态县区2个、生态乡镇6个。

　　（七）突出改善民生，推动社会事业繁荣进步。

　　坚持市级新增财力75％以上、县级新增财力60％以上用于民生事业，加快构建均等化、全覆盖、可持续的基本公共服务体系，推动民生质量不断提升、社会事业全面进步。

　　全力办好十件民生实事。今年投入86.5亿元，集中力量办好底线民生、教育、医疗卫生、困难群体、文化体育、住房、公共交通、生态、福利、法律服务等十个方面61项民生实事（具体项目见附件）。细化责任分工，强化责任落实，加大工作力度，确保全面完成各项任务。

　　持续深化文明城市创建。深入推进“好人之城”建设，推动社会主义核心价值观根植社会、融入生活。加大文明城市、文明村镇、文明单位创建力度，广泛开展文明交通、文明旅游、网络文明传播等文明实践活动。加强未成年人思想道德建设，加快筹建未成年人综合素质教育基地。完善志愿服务制度和志愿服务站建设，加快形成“10分钟志愿服务圈”。

　　推动教育事业优先发展。继续抓好义务教育均衡发展示范项目建设，改扩建、新建中小学校22所，新增学位17845个。力争义务教育阶段民办学校标准化、幼儿园规范化覆盖率90％以上。启动实施随迁子女积分入学办法。提高残疾儿童少年入学率，实施高中阶段残疾学生免费教育。申办惠州工程职业技术学院，支持惠州学院等高等院校加快发展。

　　大力发展医疗卫生事业。推进中心医院总部建设和第一人民医院扩建工程建设。免费为18万名妇女进行“两癌”筛查。继续抓好县级公立医院改革。加强疾病预防控制，提高公共卫生服务水平。加快推进医疗卫生电子信息化建设。完善基层医改运行新机制，扶持发展镇街卫生院、村卫生站、社区卫生服务中心，加强全科医生培训，提高基层医疗机构诊疗能力。

　　加快发展文化体育事业。完成第二批国家公共文化服务体系示范项目建设。创建2个省首批公共文化服务示范项目。继续做好国家历史文化名城申报和保护工作。抓紧修复东坡祠，抢修宾兴馆，推进博罗银岗窑址公园建设。加快启动建设市工人文化宫。按省一级以上标准推进镇街文化站达标升级，按　“五个有”标准完善村居文化室设施。办好第六届（惠州）东坡文化节等活动。广泛开展全民健身运动，完成120个自然村的农民体育健身工程建设。组队参加第十四届省运会，办好中华龙舟大赛等体育赛事。

　　加强就业和社会保障工作。新增城镇就业人员7万人，转移农村富余劳动力1万人。执行社保缴费基数新标准。继续提高城乡居民医保财政补助标准，医保基金年度最高支付限额提高到40万元。完善重特大疾病医疗救助、商业保险大病二次补偿制度。推进社会养老服务体系建设。进一步做好优抚安置、特困人员供养等工作，大力发展社会福利、慈善事业。建设公共租赁住房1160套，新增低收入住房保障家庭租赁补贴930户。完成华侨农场危房改造1037户、城市棚户区改造950户，推进农村危房改造。加快建设省失独家庭养老基地，抓紧迁建市救助管理站。

　　完成扶贫“双到”工作任务。加大精准扶贫力度，确保57个省重点帮扶村实现村集体经济年收入达10万元以上、贫困户年人均纯收入达8000元以上，确保扶贫“双到”三年总考核取得优秀成绩。

　　全力推进平安惠州创建。深入实施“平安细胞”工程，推动“全警大巡防”向“全民大群防”升级。深入开展“3＋X”专项行动，全力推进“无毒创建”活动。创新信访工作机制，做好矛盾纠纷预防化解，及时解决群众合理诉求。强化安全生产　“一岗双责”，深化重点行业领域“打非治违”和专项治理，坚决预防和遏制重特大事故。切实抓好食品药品安全监管，101家学校食堂安装　“阳光厨房”视频监控系统，99所农村义务教育学校食堂实行收回管理、自主经营。抓好产品质量管理和价格监督检查，依法打击走私贩私、制假售假、盗版侵权、传销和变相传销，切实维护市场经济秩序。

　　统筹发展社会各项事业。深入开展新时期爱国卫生运动，确保顺利通过国家卫生城市复检。继续抓好人口计生工作，保持全省先进。深化双拥共建，全力争创全国双拥模范城“五连冠”、广东省双拥模范城“七连冠”。积极发展妇女儿童、青少年、残疾人、红十字等事业。继续抓好国防动员、外事侨务、港澳事务、防震减灾、老区建设、民族宗教、三防、消防、应急、统计、编制、台务、仲裁、方志、档案、气象、移民、科普等工作，推动社会各项事业全面进步。

　　（八）突出依法行政，加快建设法治政府。

　　认真贯彻落实党的十八届四中全会、省委十一届四次全会精神，坚持在党的领导下、自觉运用法治思维和法治方式，改进作风，提升效率，勇于担当，促进政府工作水平全面提升。

　　加强民主法治建设。贯彻落实进一步加快法治惠州建设实施意见，深入推进依法治市。全面落实市委决策部署和人大决议决定，更加自觉接受人大及其常委会的法律监督、工作监督和人民政协的民主监督、人民群众的舆论监督，健全重大事项向人大及其常委会报告和向政协通报制度，广泛听取民主党派、工商联、无党派人士和人民团体的意见，认真做好议案、提案、建议办理工作。开展法治文化建设和宣传教育，争创全国　“六五”普法先进市。完善“法制副主任”、“法制副校长”制度，探索推行“法制副厂长”制度。健全司法救助机制，开展法律援助便民服务。积极做好申报工作，力争成为《立法法》修订后全省第一批行使地方立法权的设区的市之一。

　　扎实推进依法行政。把依法行政贯穿于政府工作全过程、各领域，坚持法定职责必须为、法无授权不可为。坚持依法、民主、科学决策，落实重大行政决策公众参与、专家论证、风险评估、合法性审查、集体讨论决定等制度。拓宽法律顾问参与政务的渠道。推进政务公开信息化。完善网络问政平台。启动行政复议庭建设。加强行政执法监督，确保严格执法、公正执法、文明执法。

　　大力提高行政效率。自觉践行群众路线，建立密切联系群众、改进作风的常态化机制。全面落实市委《关于进一步提高效率狠抓落实的意见》，大力推行　“一线工作法”，着力提高决策部署落实效率、行政审批效率、机关运转效率、为民便民办事服务效率，努力营造优质高效的政务环境。

　　切实做到廉洁从政。加强纪律建设，严守党的政治纪律和政治规矩。严格执行中央八项规定，进一步贯彻国务院“约法三章”。落实党风廉政建设“一岗双责”，深化惩防腐败体系建设。加强行政监察和审计监督。严格落实党政机关厉行节约反对浪费条例，严控“三公”经费，最大限度压缩行政开支，把更多财力投入民生事业，以政府过紧日子让群众过好日子。

　　在做好各项工作的同时，深入调查研究，科学论证分析，充分集中民智，认真做好“十三五”规划编制工作，为惠州未来五年的科学发展绘制高标准、高质量、高水平的宏伟蓝图。

　　各位代表、同志们，时代召唤我们励志前行，责任要求我们勇于担当。让我们紧密团结在以习近平同志为总书记的党中央周围，在省委、省政府和市委的坚强领导下，奋发进取，奋勇争先，奋力拼搏，全力推动惠州尽快进入珠三角第二梯队，谱写科学发展的惠民之州建设新篇章！
